# Alpha-Band Rhythms in Visual Task Performance: Phase-Locking by Rhythmic Sensory Stimulation

**DOI:** 10.1371/journal.pone.0060035

**Published:** 2013-03-29

**Authors:** Tom A. de Graaf, Joachim Gross, Gavin Paterson, Tessa Rusch, Alexander T. Sack, Gregor Thut

**Affiliations:** 1 Department of Cognitive Neuroscience, Faculty of Psychology and Neuroscience, Maastricht University, Maastricht, The Netherlands; 2 Maastricht Brain Imaging Center, Maastricht, The Netherlands; 3 Centre of Cognitive NeuroImaging, Institute of Neuroscience and Psychology, University of Glasgow, Glasgow, United Kingdom; 4 Department of Psychology, Ludwig Maximilians University Munich, Munich, Germany; Radboud University Nijmegen, The Netherlands

## Abstract

Oscillations are an important aspect of neuronal activity. Interestingly, oscillatory patterns are also observed in behaviour, such as in visual performance measures after the presentation of a brief sensory event in the visual or another modality. These oscillations in visual performance cycle at the typical frequencies of brain rhythms, suggesting that perception may be closely linked to brain oscillations. We here investigated this link for a prominent rhythm of the visual system (the alpha-rhythm, 8–12 Hz) by applying rhythmic visual stimulation at alpha-frequency (10.6 Hz), known to lead to a resonance response in visual areas, and testing its effects on subsequent visual target discrimination. Our data show that rhythmic visual stimulation at 10.6 Hz: 1) has specific behavioral consequences, relative to stimulation at control frequencies (3.9 Hz, 7.1 Hz, 14.2 Hz), and 2) leads to alpha-band oscillations in visual performance measures, that 3) correlate in precise frequency across individuals with resting alpha-rhythms recorded over parieto-occipital areas. The most parsimonious explanation for these three findings is entrainment (phase-locking) of ongoing perceptually relevant alpha-band brain oscillations by rhythmic sensory events. These findings are in line with occipital alpha-oscillations underlying periodicity in visual performance, and suggest that rhythmic stimulation at frequencies of intrinsic brain-rhythms can be used to reveal influences of these rhythms on task performance to study their functional roles.

## Introduction

Rhythms are ubiquitous in biological systems. From the sniffing of rodents [1] to the dynamics of the human attention system [2], temporal regularity is a fundamental property for organisms. While we have been aware of such periodicity in the brain [3], a number of recent studies has emphasized that rhythmicity is also apparent in behavior.

A single event can ‘reverberate’ in perceptual systems, leading to an oscillatory pattern in visual task performance time-locked to the event. For instance, one sound, when predictive of upcoming visual events, can lead to a cyclic pattern in subsequent visual target detection [4] and visual cortex reactivity [5]. A single visual flash, presented in one hemifield to capture attention, results in cyclic patterns of visual detection performance in this hemifield, and opposing (anti-phase) detection performance in the opposite hemifield [6]. Multiple events, if presented in a stable rhythm, have similar periodic effects, since rhythmic trains of events generally benefit processing of subsequent stimuli, if these are in phase with the preceding train [7]–[11]. Even continuous streams of multiple but non-periodic (random) events can be associated with illusory flicker perception at a specific frequency (see [12] for ‘perceptual echoes’). Such findings suggest a fundamental role for periodicity in perception and attention.

At the same time, oscillations in the brain have been linked to perception, attention and exploratory behavior. Sensory detection performance [13]–[15] and saccadic latency [16], [17] depend on the momentary phase of ongoing brain oscillations. Perception depends on oscillatory phase as a function of the power of these oscillations [14], and power and phase of ongoing oscillatory brain activity can be modulated by rhythmic sensory stimulation [9], [18]–[20], as can perception (see above; [7]–[11]); a process linked to and amenable to attention [9] and involving phase-locking of brain oscillations to the rhythmic sensory events [9], [21]. Finally, many of the cyclic patterns in visual performance reviewed above are in the frequency range of brain oscillations [5], [6]. Collectively, these findings raise the hypothesis that perception is closely linked to oscillations in the brain (see e.g. [22]). They also suggest that sensory stimulation may be used to study the link between perception/attention and oscillations via phase-locking of the two measures.

Here, we aim to test these hypotheses for a prominent rhythm of the visual system; the occipito-parietal alpha-oscillation (8–12 Hz). This brain rhythm has been suggested to be perceptually relevant, because modulated in both amplitude and phase by attention [23]–[28] and related to perception [14], [21], [27], [29]–[32]. We here consider the link between the occipital alpha-rhythm and perception/attention by presenting rhythmic visual events at a frequency centered in the alpha band (around 10 Hz) and probing its consequences on subsequent visual performance. Such rhythmic, 10 Hz visual stimulation has been shown to enhance occipital-parietal alpha power [19], [33], [34]. This oscillatory response enhancement is frequency-sensitive (i.e. dominant for visual stimulation at 10 Hz relative to stimulation at adjacent (flanker) frequencies [19]), and therefore likely to reflect a resonance response of the visual system [19]. This raises the question whether the alpha-power enhancement by 10 Hz-stimulation is due to the promotion of naturally occurring, perceptually relevant alpha oscillations (possibly through their phase-locking; corresponding to “entrainment”). If so, 10 Hz-stimulation should have a number of testable consequences on visual performance measures, with these predictions resting on current models on the role of alpha oscillations [35]–[37].

These predictions are: Firstly, that rhythmic stimulus trains at alpha frequency (10 Hz) should disproportionally influence visual/attentional performance relative to stimulation at flanker frequencies outside the alpha-band (i.e. should have alpha-specific effects), supporting the view that alpha-rhythms and perception are intimately linked [35], [38]. In terms of direction, one would expect this alpha-specific effect to interfere with visual/attentional performance in line with the proposed inhibitory role of alpha rhythms [36], [37]. Secondly, after rhythmic alpha-stimulation, visual task performance should oscillate over time with alpha-frequency (periodicity in perception), supporting models that perception depends on (alpha) phase [37], [39]. Thirdly, these behavioral alpha-band oscillations should correlate in precise frequency with naturally occurring alpha oscillations in the brain, which would more firmly link brain oscillations to cyclic patterns in visual performance measures and speak in favor of their causal implication in perception.

In the current study, we tested these three predictions in two experiments using rhythmic stimulation protocols inspired from [7] and [10] (see Methods for details), in which rhythmic events precede an imperative visual target. In experiment 1, we examined visual performance after rhythmic visual stimulation at 10.6 Hz (in the alpha-band) in comparison to control (flanker) frequencies below and above this band (3.9 Hz, 7.1 Hz, 14.2 Hz, 17 Hz). Crucially, the rhythmic stimulation protocol we employed ([7], [10]) foremost engages attention [7], [11], [25]. This is because the rhythmic events cue for a likely time point and spatial position of forthcoming events in the event sequence [7], [10], [11], [21], [25]. Here, we presented targets either at this cued (i.e. congruent) or at an alternative (i.e. incongruent) location. This is expected to lead to enhanced perception accuracy at congruently as compared to incongruently cued positions; an attention effect well-established for a wide range of cueing frequencies (1.8 Hz in [7], 2.5 Hz in [11], [25], 12.1 Hz in [14], [21]), henceforth referred to as ‘cueing benefit’. This cueing benefit was therefore our starting point, or baseline, relative to which we assessed our predictions in experiment 1 (i.e. diverging behavioral effects for alpha-band cueing, i.e. counteracting the cueing benefit, due to entrainment of inhibitory brain rhythms oscillating in this frequency band). In experiment 2, we more directly tested the prediction that alpha-band rhythmic cueing would lead to cycling visual task performance, presumably in coherence with underlying alpha oscillations in the brain. To this end, we correlated individual fluctuations in visual task performance with resting-state brain oscillations, and also evaluated cycling visual task performance after rhythmic cueing by an alpha-subharmonic frequency (5.3 Hz).

## Methods

### Participants

For experiment 1, a total of 22 participants volunteered. Two were authors of this paper (T.R., T.A.G.), 20 were students at Glasgow University, receiving course credits for participation. One participant was excluded due to outlier performance (reaction times >2.5 standard deviations (SD) above group average, target discrimination accuracy >2.5SD below group average), leaving a total of 21 participants (5 male, 22±3 yrs old, 4 left-handed). For experiment 2, 20 participants volunteered. Two were authors of this paper (T.R., T.A.G.). 18 were students at Glasgow University, compensated with course credits. Two participants were excluded on the basis of outlier performance (S1: reaction times >2.5SD above group average, S2: target discrimination accuracy >2.5SD below new group average). In total 18 subjects were included in the analysis (10 male, 23±4 yrs, 3 left-handed). All subjects had normal or corrected-to-normal vision.

### Ethics Statement

This work was approved by the ethics committee of the institution where measurements took place (Centre of Cognitive NeuroImaging, Institute of Neuroscience and Psychology, University of Glasgow, United Kingdom). All subjects provided written informed consent.

### Rhythmic Stimulation Paradigms

We implemented two rhythmic stimulation paradigms: a stationary as well as an apparent motion entrainment/cueing paradigm (‘flicker’ versus ‘motion’ cues). We call these paradigms interchangeably ‘entrainment’ or ‘cueing’ as the rhythmic stimulus train (the ‘entrainers’) cues for the upcoming visual target. Our design for ‘flicker’ entrainment (cueing) was inspired from Mathewson et al. [10], who showed that rhythmically presenting a visual annulus at one position benefits perception of an upcoming visual target at this position, when targets were presented in phase with the preceding train (possibly constituting a baseline attentional benefit). Only a few rhythmic pre-target cues (n = 2–8) sufficed to benefit perception [10]. Our design for ‘motion’ entrainment (cueing) was inspired from Doherty et al. [8] who showed that a visual disk rhythmically crossing the computer monitor benefits subsequent target perception if the target position and/or timing were predictable from the entrainment cues (inferred to represent an exogenous attentional benefit, see e.g. [11]). Importantly, flicker cues are also associated with a resonance (entrainment) phenomenon in oscillatory brain response at 10 Hz [19], with potential consequences on perception (our hypothesis). The motion entrainment condition was implemented in order to test to what extent perceptual consequences of entrainment at alpha frequency would be restricted to retinotopically specific mechanisms – i.e. would only be observed with flicker stimulation – or also applies to rhythmic motion stimuli, in which entrainers move across the visual fields. In our modified versions of these paradigms, ‘flicker’ and ‘motion’ cueing were implemented as follows.

**Figure 1:**
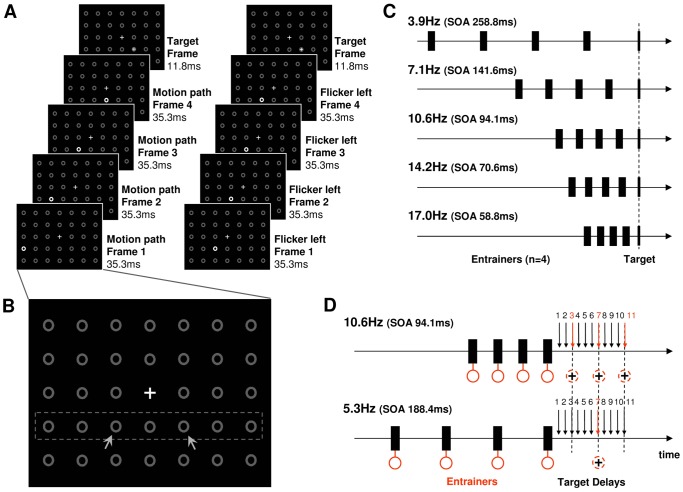
Stimuli and Design. **A.** A 5×7 array of annuli and a fixation cross were presented at all times. Rhythmic cueing consisted of the briefly (35.3 ms) brightening (‘flashing’) of annuli in a predictable sequence. In ‘motion entrainment’ (left), 4 annuli would flash along a spatial path, starting either with the left-most (example) or right-most annulus, and ending with the central annulus below the fixation cross. A visual target was then presented in the centre of the next annulus of the motion path (cued/congruent location) or in the annulus that flashed before (incongruent location). In ‘flicker entrainment’ (right), the same annulus would flash 4 times. The flashing annulus could be at the left (example) or right target locations. Depending on whether the target location coincided with the cued location, cueing was either congruent or incongruent. For both forms of entrainment, targets appeared at congruent and incongruent positions with equal likelihood, thus cueing was spatially non-predictive. **B.** Enlarged view of the stimulus array, indicating for illustration purposes by rectangle the row of annuli that were involved in motion/flicker cueing, and by arrows the two possible target locations. **C.** Design of Experiment 1 testing five cueing frequencies. Visual targets were always ‘in-phase’ with the cue-train. **D.** Design of Experiment 2 testing two cueing frequencies and 11 stimulus onset asynchronies (from last cue to visual target). These covered three cycles at alpha frequency.

A matrix of 5×7 annuli and a central fixation cross were presented at all times on the screen (grey on black background, Fig. 1A and B). We refer to these annuli as ‘placeholders’. Entrainers consisted of placeholders briefly ‘lighting up’, or ‘flashing’. During *flicker entrainment*, one placeholder would flash four times consecutively, before a visual target was presented (see Figure 1A, right for one example trial). Only placeholders of two positions could flash (diagonal to the lower right or lower left of the fixation cross, marked in Fig. 1B for illustration purposes by arrows), and targets could appear either in the center of the placeholder that flashed, or in the opposite hemifield with 1∶1 probability. Target position could thus be *congruent* or *incongruent* relative to cued position, but importantly, flicker cues were spatially non-predictive as to target positions. During *motion entrainment*, four placeholders of the row of circles below the fixation cross (marked in Fig. 1B for illustration purposes by a rectangle) would flash in succession, either starting with the right-most circle and ending with the central circle directly underneath the fixation cross, or starting with the left-most circle and ending with the same central circle (see Figure 1A, left for one example trial). This was followed by a target presented in the adjacent placeholders, left or right from the last entrainer with 1∶1 probability (i.e. in or out-of motion path, and at the same positions as the targets of the flicker condition, marked in Fig. 1B). Thus, again target positions could be *congruent* or *incongruent* relative to the direction of motion cueing, but motion cues were spatially non-predictive as to target positions.

We foremost expected an advantage of visual task performance at cued versus uncued positions (in line with [7], [10]). Entrainers flashing at/towards the left or right position (flicker/motion cues) should benefit visual performance at cued (left or right) location. Note that our task discouraged endogenous cueing (by rendering target appearance at cued and uncued positions equally probable), and emphasized exogenous cueing to specific positions (by the spatiotemporal structure of the rhythmic stimulus trains). Irrespective of the mechanisms employed, we expected (1) this cueing benefit to be disproportionally affected by alpha-entrainment/cueing, (2) visual task performance to cycle at alpha-frequency, and (3) periodicity in perception to correlate with periodicity in brain rhythms, if brain oscillations in the alpha-band indeed play a role in perception.

### Experimental settings, stimulus parameters, and task

Participants were seated 0.30 m in front of a CRT monitor (refresh rate 85 Hz). Viewing distance was kept stable using a chin rest. In an initial training phase, participants were familiarized with the task, and target salience was individually adjusted to approximately 80% target discrimination accuracy to avoid ceiling effects. The training phase consisted of a subset of trials but included all conditions tested in the main experiment, and was repeated until stable performance was reached.

The annuli of the placeholder matrix were 1.5 cm in diameter, spaced apart 5.3 cm horizontally and 5 cm vertically. For entrainment, annuli briefly changed (flashed) from grey to white for three frames (35.3 ms). Visual targets consisted of an ‘x’ or ‘+’ and were always presented for the duration of one frame only (11.8 ms). Participants were asked to fixate the central fixation cross at all times. The task was to indicate by means of button presses whether a ‘+’ or an ‘x’ sign (rotated ‘+’) appeared on screen.

### Testing for alpha-specificity of effects (experiment 1)

In experiment 1, we evaluated the effects of congruency (cued vs. uncued position), and rhythmic cueing type (flicker vs. motion). To test for frequency-specificity, we implemented entrainment at five frequencies (3.9 Hz, 7.1 Hz, 10.6 Hz, 14.2 Hz, 17 Hz, Fig. 1C). Note that for all conditions of Experiment 1 (motion vs. flicker, congruent vs. incongruent, 5 frequencies), visual targets followed ‘in phase’ with the entrainment, i.e, a regular interstimulus interval was used for presenting the four entrainers and the subsequent targets (Fig. 1C) such that the onset of the visual target coincided with the onset of what would have been a fifth entrainer in the train. All conditions were presented in random order across trials in five runs. A total of 1200 trials were sampled, resulting in 60 trials per condition cell per participant.

### Testing for periodicity in visual performance measures (experiment 2)

In experiment 2 only motion entrainment was implemented. Aside from testing for congruency effects (see above), we tested entrainment at two frequencies (10.6 Hz, 5.3 Hz) and varied stimulus onset asynchrony (SOA) between the fourth (last) entrainer and the visual target (Fig. 1D). We tested 11 SOAs, starting from 47.1 ms, up to 282.4 ms, in steps of 23.5 ms (2 frames). Note that the SOA at which the visual target was in-phase with the entrainers is at 94.1 ms for 10.6 Hz-cueing and at 188.2 ms for 5.3 Hz-cueing (3^rd^ versus 7^th^ tested SOA, Fig. 1D, see dashed annuli), and that our range of SOAs covered three cycles of an alpha-oscillation, allowing for detection of a possible cyclic pattern in behavioral performance at alpha-frequency. All conditions were presented in random order across trials in seven runs. A total of 1232 trials were sampled, resulting in 28 trials per condition cell per participant.

### Testing for a link to brain rhythms (experiment 2b)

To evaluate the relationship between the cyclic pattern in visual performance and actual brain oscillations, we measured resting-state alpha oscillations (eyes open and closed, five minutes) using a 248-magnetometer whole-head MEG-system (MAGNES® 3600 WH, 4-D Neuroimaging) in fourteen participants of experiment 2 who were available for this follow-up measurement. One subject who could not be measured with MEG was measured with EEG instead (8 parieto-occipital electrodes), bringing the total number of subjects to 15. For simplicity we continue to refer to “MEG measurements” below.

### Preprocessing behavioral data

Only trials with reaction times between 200 and 1200 ms were included to remove outliers in both experiments. Accuracy (proportion correct) served as the dependent variable of interest.

### Analysis Experiment 1

We implemented a full within-subjects design with factors Rhythmic cueing type (motion, flicker)×Congruency of cueing (congruent, incongruent)×Frequency of cueing (5 levels). Accuracy (proportion correct) per condition was subjected to repeated-measures Analysis of Variance (RM-ANOVA). Results were further explored using follow-up RM-ANOVAs or 2-tailed paired-samples t-tests where appropriate, as indicated in the Results section.

### Analysis Experiment 2

In experiment 2, we focused on the temporal profile of visual task performance over Delays (11 SOAs) between visual target onset and the last cue. To evaluate whether a cyclic pattern in visual task performance was apparent, we applied curve-fitting procedures in custom software using robust nonlinear least-squares fitting in MATLAB. We analyzed group-averaged accuracy for all conditions separately (10.6 Hz and 5.3 Hz, spatially congruent and incongruent locations), after linearly detrending the data to remove linear effects across SOA and retain any cyclic patterns around the mean. We then fitted both 10 Hz and 5 Hz cosine curves to the data (fixed frequency, variable phase). R-squared values of the group mean data were statistically evaluated using bootstrapping. To this end, labels of the 11 SOAs were randomly permuted over 2500 iterations, and a model cosine (10 Hz or 5 Hz) was fitted to the resulting behavioral pattern each time, generating a null distribution of 2500 R-squared values. The R-squared value obtained from the actual data was related to this created null-distribution to evaluate whether it fell in the top-95^th^ percentile. If so, this by definition indicated that the model cosine significantly explained variance in the group data.

Aside from the above-described group analysis, we performed a secondary analysis in which we fitted model cosines to the behavioral data of individual participants, obtaining two R-squared values for each participant, cueing frequency, and congruency level. These two R-squared values resulted from fitting a flexible 10 Hz cosine (freely ranging between 8–12 Hz) and a flexible 5 Hz cosine (freely ranging between 3–7 Hz); this flexibility allowing for inter-individual variability in peak frequency. A second-level analysis then evaluated whether 10 Hz cosine models fitted the individual data better than 5 Hz cosine models (one-tailed paired-samples t-test between 10 Hz-based versus 5 Hz-based R-squared values).

### Analysis Experiment 2b

Using standard Fourier transforms over parieto-occipital sensors of the recorded MEG data, we could identify a clear peak in the alpha band (8–12 Hz) for each participant (individual alpha-frequency). We then fitted a model alpha cosine (using robust nonlinear least-squares fitting) to the behavioral 10.6 Hz-data of each of the 15 participants to extract the frequency that best reflects the individual behavioural patterns (fitting based on freely-ranging frequencies, i.e. 7–13 Hz). Note that on the group level, a cosine always better fitted the detrended than the original data. On the individual level, detrending distorted the behavioural data in some participants who showed no strong trend but had outliers at the first or last position. Outliers affected the (non-robust) detrending but not the (robust) curve fitting. To avoid biased results, fitting was performed on both original and detrended data and the best-fitting result was used to extract the individual behavioural alpha peak. We then tested for a positive relationship between frequencies in behavioural and MEG data (Pearson correlation, one-tailed testing) to compare behavioral patterns with intrinsic brain oscillations.

## Results

### Alpha-specific breakdown of cueing benefits (experiment 1)

We investigated spatial cueing benefits from attentional mechanisms associated with rhythmic (entrainment) cues as a function of cueing frequency in two entrainment conditions (flicker vs. motion, Fig. 1A–B). We tested this by assessing visual task performance (target discrimination accuracy) at the first time-point in phase with the preceding entrainer-cues (Fig. 1C). The overall repeated-measures ANOVA on visual discrimination accuracy with factors Rhythmic Cueing Type (flicker vs. motion), Congruency of cueing (congruent vs. incongruent), and Frequency of cueing (3.9, 7.1, 10.6, 14.2 vs. 17 Hz) showed a main effect of Rhythmic Cueing Type (F[1,20] = 7.01, P<0.05). Performance was weakly but significantly better with motion cueing (accuracy = 0.82) than flicker cueing (accuracy = 0.80). This is likely due to a forward masking of the visual target by the final cue, which occurs in the flicker, but not in the motion condition. Importantly, Rhythmic Cueing Type did not interact with any other condition (3-way interaction: F[4,80] = 0.74, P = 0.571, 2-way interaction with Congruency: F[1,20] = 0.12, P = 0.728, 2-way interaction with Frequency: F[4,20] = 1.18, P = 0.325). In other words, all the effects we are about to describe were statistically not different for both types of cueing, and therefore likely to be independent of masking.

**Figure 2:**
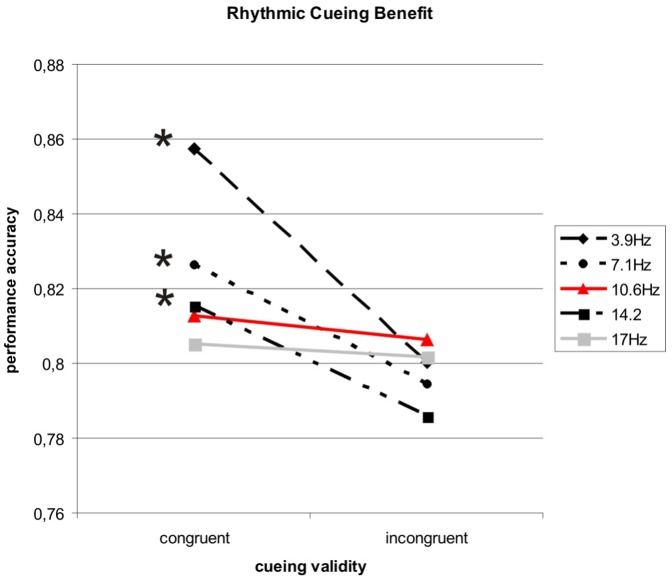
Results Experiment 1. Group averaged accuracies for target discrimination at congruent and incongruent locations as a function of the five cueing frequencies (exploring a significant Congruency×Frequency interaction). In spite of spatially non-predictive cueing, significant cueing benefits (higher accuracy at cued relative to uncued locations) were observed for cueing at 3.9 Hz, 7.1 Hz, and 14.2 Hz but broke down for intermediate 10.6 Hz cueing in the alpha-band (8–12 Hz). At the highest frequency of 17 Hz, rhythmic cueing no longer led to cueing benefits. *: significant cueing benefits at p<0.05.

The overall ANOVA revealed a main effect of Congruency of cueing (F[1,20] = 8.44, P<0.01), with better visual task performance at cued than uncued locations (cueing benefit), despite rhythmic cueing being spatially non-predictive regarding upcoming target position, and therefore possibly due to exogenous attention mechanisms driven by the cues. Importantly, this effect was dependent on the Frequency of cueing (F[4,80] = 3.69, P<0.01), showing that attentional cueing was not equally effective over all frequencies. Figure 2 illustrates visual task performance for congruent and incongruent cueing across all frequencies, collapsed over flicker and motion cueing (due to absence of a 3-way interaction, see Table 1 for noncollapsed data across all conditions). A cueing benefit with better performance at cued than uncued location is observed for rhythmic cueing at 3.9 Hz (t-test for congruency effect: P<0.001), 7.1 Hz (P<0.05) and 14.2 Hz (P<0.05), hence a broad range of frequencies, but not for the 10.6 Hz (P = 0.662) or the 17 Hz (P = 0.783) condition. There is thus a discontinuity of cueing benefit with alpha-stimulation compared to adjacent flanker frequencies. In line with our expectations, we therefore found a frequency-specific break-down of cueing benefits at 10.6 Hz stimulation.

**Table 1:** Data of experiment 1 (outliers removed), split across conditions.

	Flicker Cueing				Motion Cueing			
	Cue Left		Cue Right		Cue Left		Cue Right	
	Target Left	Target Right	Target Left	Target Right	Target Left	Target Right	Target Left	Target Right
3.9 Hz	0.858 [0.076]	0.781 [0.090]	0.777 [0.091]	0.845 [0.079]	0.832 [0.082]	0.867 [0.074]	0.864 [0.075]	0.819 [0.084]
7.1 Hz	0.806 [0.086]	0.806 [0.086]	0.789 [0.089]	0.840 [0.080]	0.795 [0.088]	0.861 [0.076]	0.799 [0.087]	0.786 [0.090]
10.6 Hz	0.804 [0.087]	0.785 [0.090]	0.797 [0.088]	0.802 [0.087]	0.804 [0.087]	0.836 [0.081]	0.804 [0.087]	0.833 [0.082]
14.2 Hz	0.805 [0.086]	0.784 [0.090]	0.757 [0.094]	0.801 [0.087]	0.825 [0.083]	0.835 [0.081]	0.816 [0.085]	0.775 [0.091]
17 Hz	0.817 [0.084]	0.785 [0.090]	0.788 [0.089]	0.762 [0.093]	0.828 [0.082]	0.823 [0.083]	0.811 [0.085]	0.801 [0.087]

Displayed are average proportion correct and [standard error of the mean].

### Periodicity in visual task performance at 10 Hz frequency (experiment 2)

In experiment 2 we tested effects of cueing at 10.6 Hz and at the first alpha-subharmonic (i.e. 5.3 Hz). If phase-locking of naturally occurring alpha oscillations drives our results, this alpha-subharmonic should have similar effects as 10.6 Hz-cueing (although possibly to a lesser extent), since the 5.3 Hz condition constitutes a ‘weak’ 10.6 Hz entrainer rhythm with every second entrainer left out. As in experiment 1, 10.6 Hz-cueing in experiment 2 did not result in a cueing benefit (P = 0.133), nor did alpha-subharmonic cueing at 5.3 Hz (P = 0.293).

**Figure 3:**
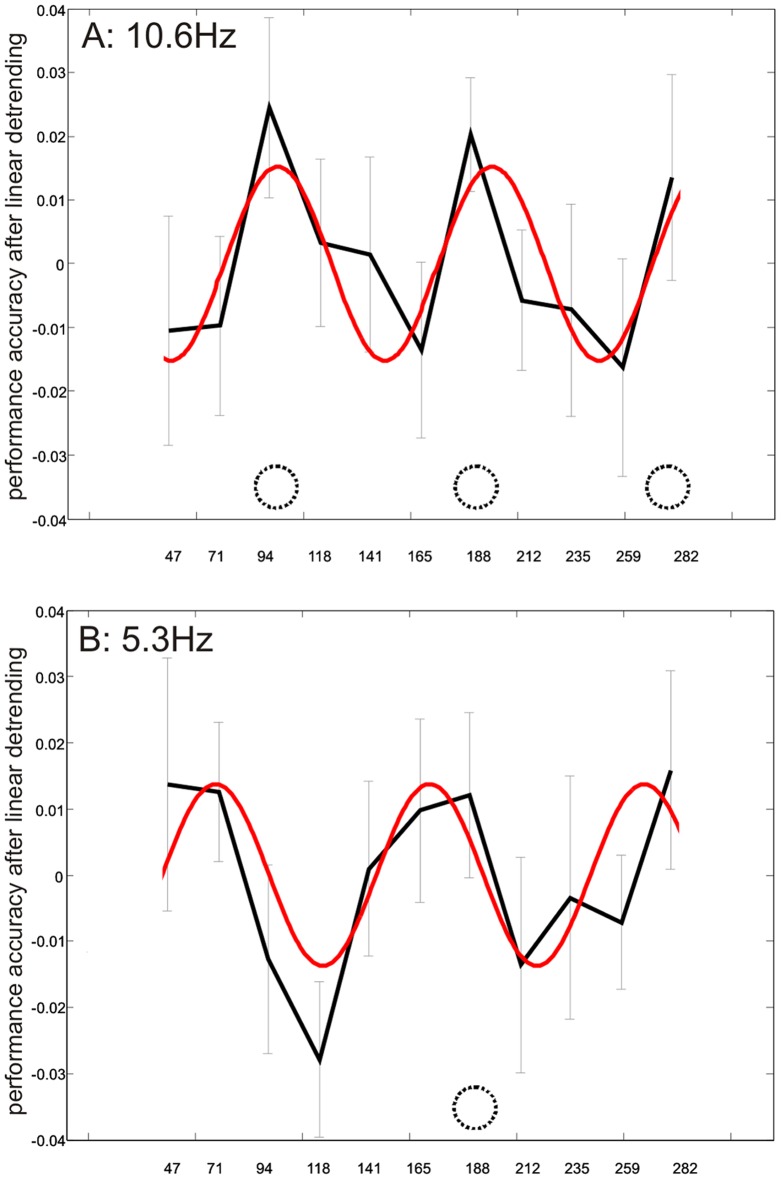
Results Experiment 2. Group averaged accuracy (linearly detrended) for target discrimination at congruent locations over 3 alpha cycles post-train following **A**) 10.6 Hz- or **B**) 5.3 Hz-cueing. The best fitting 10 Hz model cosines are superimposed. For both 10.6 Hz and 5.3 Hz (subharmonic) congruent cueing, we found recurrent peaks and troughs which were significantly fitted by a 10 Hz cosine function. Thus, a cyclic pattern at alpha-frequency is apparent in visual task performance. Error bars reflect standard error of the mean after removal of baseline between-subject variance (within-subject error bars: [49]).

If 10.6 Hz-cueing indeed leads to promotion of underlying alpha-oscillations in the brain (by phase-locking) and the phase of this oscillation is functionally relevant for visual task performance, then visual task performance should cycle over time post-train at alpha-frequency, in coherence with the entrained alpha-oscillation. We tested target discrimination over a window of ∼300 ms after the last entrainer (3 alpha cycles, 11 SOAs), using only motion entrainment. Figure 3 illustrates the time-course of visual task performance after 10.6 Hz- and 5.3 Hz-cueing for spatially congruent targets (after linear detrending, see Table 2 for original data), with the best-fitting 10 Hz cosine models superimposed.

**Table 2:** results of experiment 2 (outliers removed), before detrending.

			47	71	94	118	141	165	188	212	235	259	282
congruent	10.6 Hz	mean	0.856	0.856	0.888	0.866	0.863	0.847	0.878	0.853	0.851	0.840	0.866
		sem [WS]	0.023 [0.018]	0.030 [0.014]	0.017 [0.014]	0.022 [0.013]	0.026 [0.015]	0.029 [0.014]	0.020 [0.009]	0.027 [0.011]	0.029 [0.017]	0.029 [0.017]	0.018 [0.016]
	5.3 Hz	mean	0.879	0.877	0.852	0.837	0.863	0.872	0.876	0.851	0.862	0.856	0.881
		sem [WS]	0.030 [0.019]	0.023 [0.011]	0.027 [0.014]	0.026 [0.012]	0.026 [0.013]	0.024 [0.014]	0.026 [0.012]	0.029 [0.016]	0.030 [0.018]	0.027 [0.010]	0.025 [0.015]
incongruent	10.6 Hz	mean	0.880	0.899	0.856	0.880	0.850	0.835	0.870	0.863	0.876	0.900	0.830
		sem [WS]	0.015 [0.013]	0.019 [0.014]	0.030 [0.018]	0.024 [0.018]	0.026 [0.014]	0.025 [0.012]	0.021 [0.010]	0.024 [0.014]	0.024 [0.013]	0.018 [0.009]	0.030 [0.019]
	5.3 Hz	mean	0.872	0.845	0.815	0.863	0.843	0.852	0.854	0.825	0.870	0.834	0.837
		sem [WS]	0.017 [0.013]	0.030 [0.013]	0.032 [0.016]	0.023 [0.014]	0.031 [0.022]	0.023 [0.014]	0.024 [0.015]	0.033 [0.012]	0.025 [0.013]	0.026 [0.012]	0.032 [0.016]

Displayed are the average proportion correct per condition, per SOA (horizontal direction). Also shown are standard error of the mean (SEM), before and [after removal of between-subject variability (within-subject: WS)].

Visual inspection clearly reveals a cyclic pattern of performance peaks after 10.6 Hz motion cueing (Fig. 3A). Moreover, the peaks in this cyclic pattern are exactly in-phase with the preceding rhythmic cues, and the periodic pattern seems to span over at least three 10 Hz cycles (i.e. presenting three recurrent performance peaks rather than only one confined to the first in-phase SOA, 94.1 ms). Curve fitting procedures and permutation tests (see Methods) revealed that a 10 Hz cosine model significantly fitted the 10.6 Hz group data (57% explained variance, bootstrapped 95%-cut-off at 53%), statistically confirming the presence of a 10 Hz cyclic pattern at the cued position. For comparison, 10 Hz cosine models could not explain performance at the incongruent position (7% explained variance: cut-off 54%), nor was performance explained by 5 Hz models at congruent (15% explained variance: cut-off 52%) or incongruent positions (31% explained variance: cut-off 53%).

Cueing at 5.3 Hz led to similar results at cued positions (Fig. 3B). Visual inspection again reveals an oscillatory pattern with 3 peaks. Statistically, a 10 Hz cosine model significantly explained performance fluctuations in the group curve in this congruent condition, despite 5.3 Hz cueing (54% variance explained, cut-off: 53%). For comparison, fitting a 5 Hz cosine wave to these data did not explain its variance (6% explained, cut-off: 52%), nor was the variance in the incongruent condition explained by 10 Hz fitting (24% explained variance, cut-off 51%) or 5 Hz fitting (10% explained variance: cut-off 49%). In short, there was more evidence for a 10 Hz than a 5 Hz induced wave in the behavioural data, despite cueing at 5.3 Hz. Moreover, just as in the 10.6 Hz cueing condition, this effect was specific to the spatially congruent condition.

While this analysis conclusively demonstrated the existence of an alpha-frequency oscillation in the behavioral results, we performed a second-level analysis on the individual behavioral data. We again fitted 10 Hz and 5 Hz cosine models, and subsequently tested whether the alpha-band cosine models could better explain individual behavioral data than the 5 Hz cosine model. The 10 Hz cosine models generally explained more variance than 5 Hz cosine models, but this difference was not significant for the incongruent cueing conditions (10.6 Hz: P = 0.23, 5.3 Hz: P = 0.28). Yet, for congruent cueing at 10.6 Hz, individual behavioral data were significantly better explained by an alpha-band cosine model than by a 5 Hz cosine model (t[17] = 3.06; P<0.01). Moreover, this difference (10 Hz-fit>5 Hz-fit) even approached significance (t[17] = 1.52, P = 0.07) for (inherently suboptimal) cueing at the alpha-subharmonic frequency of 5.3 Hz. Thus, the results from second-level statistics performed on individual subjects largely converge with the findings of our primary analysis on the group data described above.

### Oscillations in visual task performance are linked to occipito-parietal brain rhythms (Experiment 2b)

**Figure 4:**
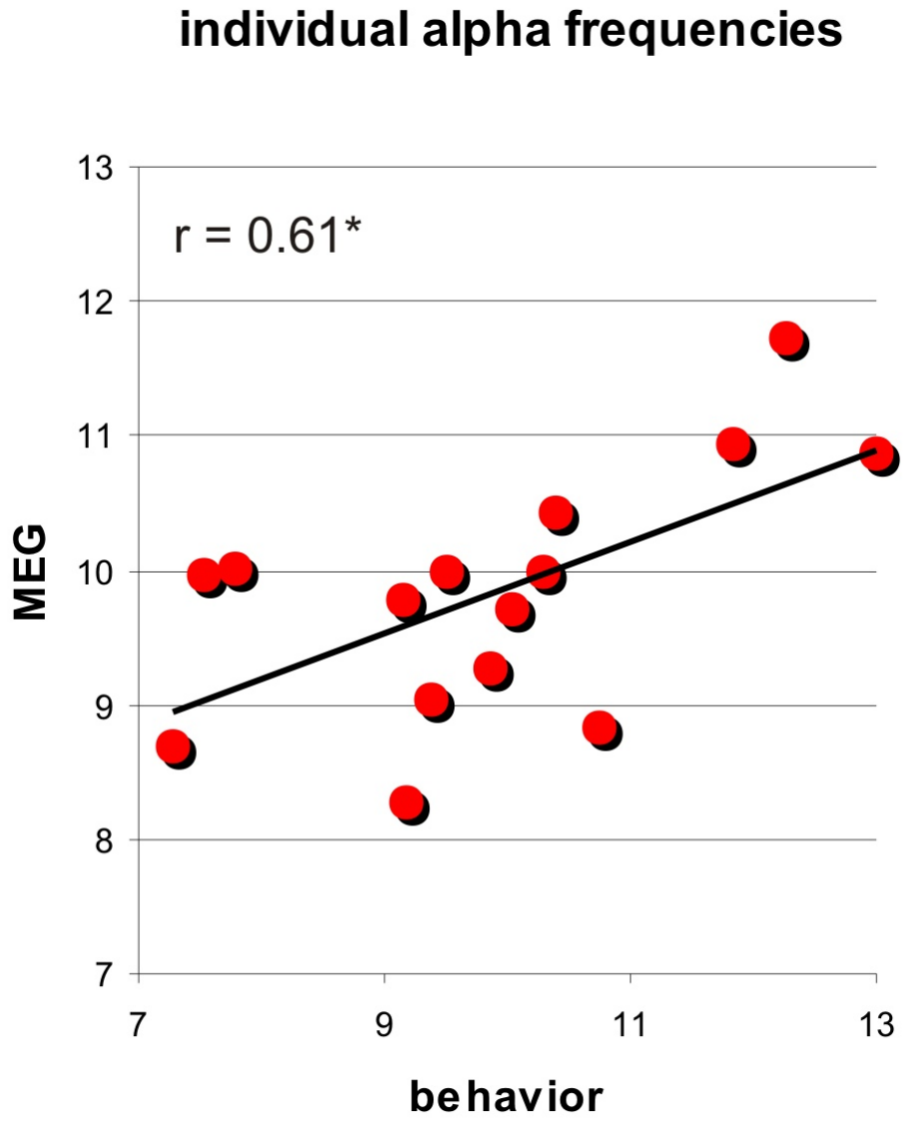
Behavior-MEG correlation. Scatterplot and linear regression analysis between frequency of rhythms in visual task performance (Experiment 2, x-axis) and MEG (recorded in a subset of participants, y-axis). The behavioural cyclic pattern showed a significant positive correlation with individual resting occipito-parietal alpha frequency. This suggests that the cyclic pattern in behaviour (shown for the group results in Figure 3) may reflect entrainment of intrinsic brain oscillations in the alpha-band.

To link periodicity in visual performance to intrinsic alpha-oscillations in the brain, we tested for a positive correlation between the best fitting frequency in individual behavioural data (cosine model, 10.6 Hz condition) and the individual alpha-frequency over occipito-parietal areas in resting-state MEG measurement (obtained for 15 participants of Experiment 2, see Methods). Figure 4 shows the resulting scatterplot and regression result. Although we used only four entrainers (and did not tune stimulation frequency to individual alpha-oscillations, i.e. fixed it to 10.6 Hz), the individual frequency in task performance significantly correlated with the frequency of the intrinsic alpha-oscillation obtained in the same participants on a different day (r = 0.61, P<0.01). This correlation confirms a link between the behavioral performance fluctuation and oscillations in the brain. Moreover, it demonstrates that the behavioral results on the group-level (Figure 3) are not due to only a small subsample of participants.

## Discussion

The results of experiment 1 reveal broadband rhythmic cueing benefits on visual target discrimination for a frequency range between at least ∼4–14 Hz (in line with previous reports, [7], [10], [11], [21], [25], discussed below), with the exception of 10.6 Hz cueing where benefits broke down. The latter frequency is centered in the alpha-range (8–12 Hz), a prominent inhibitory rhythm of the posterior brain [36]. In the second experiment, we reproduced the break-down of cueing benefits for 10.6 Hz-stimulation and subharmonical (5.3 Hz) cueing, and revealed an alpha-rhythm in visual task performance over 3 cycles post-cueing, which was also present after 5.3 Hz entrainment, and correlated in frequency with individual resting alpha-oscillations over occipito-parietal areas. The cyclic pattern in visual task performance at frequencies of intrinsic rhythms following stimulation with these frequencies not only suggests that these oscillations have been entrained [19], but also that they are functionally relevant and that entrainment paradigms can be used to study the role of oscillations in behaviour [20], [22].

### Insights into the role of oscillatory brain activity in attention and perception from alpha-specificity and alpha-periodicity of behavioural effects to rhythmic visual stimulation

Experiment 1 shows that the entrainment/cueing effects on target discrimination are frequency-specific. Cueing benefits, which are generally broadband, can break down for stimulation at alpha-frequency (10.6 Hz), or its subharmonic (5.3 Hz). The most likely explanation of these results is that two competing mechanisms are taking place; a benefit from attentional deployment by rhythmic cueing on the one hand, which is abolished by consequences of rhythmic stimulation at alpha frequency on the other hand. Both competing mechanisms are likely to involve opposite changes in perceptually relevant alpha-oscillations in occipito-parietal cortex, as identified in previous research (see e.g. [19] vs. [25]). It is well-established that the perceptual benefit from endogenous attention deployment goes along with a down-regulation in occipito-parietal alpha-power (8–12 Hz) (e.g. [26], [27]); an effect that has also been reported for rhythmic motion cueing paradigms engaging automatic attention processes [11], where downregulation of parieto-occipital alpha-power is maximally time-locked to the expected target onset (shown for 1.25 Hz- and 2.5 Hz-cueing in [25]). In contrast, alpha-frequency stimulation may lead to an up-regulation of alpha-power [19], [33], [34], e.g. by phase alignment of ongoing posterior alpha oscillations (see our hypothesis). These two mechanisms (alpha-downregulation by attention, alpha-upregulation by entrainment) are likely to counteract – thus abolishing the well-established attentional benefits from cueing paradigms. This would help to explain why 10.6 Hz-cueing did not convey a benefit in our study, while benefits for directly adjacent flanker frequencies outside the alpha-band (7.1 Hz, 14.2 Hz) were evident (for an explanation why there is no cueing benefit at 17 Hz please see below: Insights into the mechanics of exogenous spatiotemporal attention processes). It is only with 10.6 Hz-cueing (or its subharmonic) that a rhythmic train should synchronize intrinsic alpha-oscillations because progressively phase-locking the oscillatory brain activity in the course of the stimulus train (corresponding to entrainment, [20]). Indeed, cueing at the alpha subharmonic frequency of 5.3 Hz also yielded no cueing benefits (experiment 2), despite strong cueing benefits at flanker frequencies (3.9/7.1 Hz, in experiment 1). Alpha-synchronization (by the rhythmic train) counteracting the task-relevant (attention-related) alpha-desynchronization needed for conferring perceptual benefits therefore form a parsimonious mechanistic account of our results. This further supports our conjecture of entrainment of brain rhythms in the alpha band. These frequency-specific effects therefore point to a special role for alpha-oscillations in visual perception.

It is of interest to note that we have recently obtained analogous results (to experiment 1) using rhythmic transcranial magnetic stimulation (TMS) at 10 Hz over occipito-parietal areas to entrain intrinsic alpha oscillations [31] (see [40] for EEG evidence of alpha-entrainment by alpha-TMS). This TMS study also included stimulation at (control) flanker frequencies, but eliminated attention effects of rhythmic trains on visual performance by sham TMS (i.e. correcting for the effects of rhythmic coil clicks, [31]). As a result, the present experiment 1 and the previous TMS study differ in visual performance at baseline (control flanker frequencies), with baseline performance of the current study being asymmetric from the start (general attentional cueing benefit at cued vs. uncued positions, against which the effects of 10.6 Hz-trains was assessed) versus baseline performance in the TMS study being the same (symmetric) in both visual fields [31]. Despite this discrepancy in baseline between the two studies, the directional change after rhythmic stimulation was equivalent for both studies. 10 Hz-TMS lead to lower visual performance in the visual field contralateral to TMS and an enhanced performance ipsilaterally, i.e. TMS lead to a visual performance asymmetry [31]. In the present study, 10.6 Hz-stimulation abolished the baseline asymmetry. When compared to stimulation at control flanker frequencies (data of 7.1 Hz and 14.2 Hz collapsed), 10.6 Hz stimulation lowered discrimination rate at cued and increased this rate at uncued locations. That is, in both cases (occipito-parietal 10 Hz-TMS and 10.6 Hz visual stimulation), rhythmic stimulation led to a suppression of perception at cued/contralateral and enhancement at uncued/ipsilateral position.

One view holds that visual brain oscillations may implement a periodic sampling mechanism for perception [2], [37], [41], [42] with enhanced visual performance at one preferred phase of the oscillatory cycle and reduced performance at the opposite phase. Yet, only a few studies we are aware of have attempted directly to entrain intrinsic alpha oscillations (reviewed in [20], see also [43] ) in order to study phase-dependence of perception over several alpha cycles. Previous reports of perceptual benefits from rhythmic cueing [7], [10], [11], [25] could generally be explained by a top-down driven cognitive anticipation process, even when cueing effects or neural correlates thereof are recurrent (show a cyclic pattern) [21], [25] because the rhythmic cueing pattern per se generates expectations for cyclic reoccurrence of events. In contrast, top-down driven anticipation cannot explain the periodicity in perception in our results (experiment 2), for two reasons. First, after rhythmic stimulation with the alpha-subharmonic 5.3 Hz, there was an alpha oscillation in visual task performance over time, whereas a top-down driven cognitive anticipation process would have yielded a 5.3 Hz pattern. Second, across participants, the individual, peak alpha frequencies in visual task performance correlated with peak alpha frequencies in resting state MEG. The intrinsic alpha oscillations seem therefore to underlie the behavioral alpha oscillations, further ruling out anticipation as a possible explanation. Our results therefore strongly suggest that a rhythmic train of visual cues at alpha frequency (10.6 Hz) can reveal a rhythmic pattern in visual task performance driven by alpha oscillation in visual areas.

### Insights into the mechanics of exogenous spatiotemporal attention processes

Previous studies implemented rhythmic cueing paradigms similar to those we employed to study the mechanisms and constraints of attention processes through examining cueing benefits across experimental manipulations [7], [10], [11], [21], [25]. Although the cueing benefit in the present study primarily served as a baseline (against which effects of possible entrainment of brain oscillations at 10.6 Hz rhythmic stimulation were assessed), we here discuss the implication of our data also in terms of attention research.

Our results demonstrate a broadband cueing benefit (∼4–14 Hz), which is in line with previous findings [7], [10], [11], [21], [25]. Behavioral effects to rhythmic flicker- or motion-cues consist of enhanced perception of targets appearing at expected time-points or positions, i.e. in temporal and/or spatial alignment with the preceding rhythmic cues, for stimulation at 1.8 Hz [7], 2.5 Hz [11], [25], and 12.1 Hz [10], [21]. In contrast to previous studies on attention using rhythmic cueing however (e.g. [7]), we did not manipulate predictability of targets at spatial positions, i.e. targets were equally likely to occur at cued or uncued locations. Although not at all spatially predictive (but fully temporally predictive), the rhythmic trains of cues did improve visual target processing at the cued relative to the uncued locations. We therefore interpret our spatial cueing benefit to result from automatic exogenous visuospatial attention mechanisms, rather than endogenous attention control. This is in line with recent findings by Rohenkohl et al. [11], [25] who showed that the rhythm in periodic visual stimulation likely activates automatic exogenous temporal attention mechanisms, while (here absent) symbolic information can affect endogenous temporal attention mechanisms. Overall, our data confirm the notion that during rhythmic cueing, temporal expectations cooperate with visual spatial attention for optimizing perceptual analysis at/towards cued locations [7] over a broadband of stimulation frequencies [10], [11], [21], [25], and that this is under automatic (exogenous) attention control [11].

In addition, our findings provide information on possible dynamic limits of these attention processes. Apart from the breakdown of benefits at alpha-rhythms, the frequency of cueing differentially affected perception in such a way as to suggest that automatic attention processes with dynamic limits are at play (highest cueing benefit at lowest frequency = 3.9 Hz, decreasing cueing benefit as frequency of stimulation increases). Towards an upper dynamic limit, spatial and temporal attention may be progressively less able to engage, disengage and reallocate to present and future events of the rhythmic event sequence to finally break off beyond this limit. Our data may indeed point towards such an upper dynamic limit which would be at 14–17 Hz (at least for our experimental setting with four entrainers), as indicated by the lack of cueing effects at 17 Hz (the highest frequency we tested). However, given our interpretation of the lack of cueing benefits at 10.6 Hz frequency (involving counteracting attention vs entrainment mechanisms), the question arises whether the lack of cueing benefit at 17 Hz could not be explained similarly. Several arguments however speak against this explanation, i.e. against the lack of cueing benefits at 17 Hz reflecting entrainment of inhibitory brain rhythms competing with an attentional benefit. Firstly, there is no evidence for a neuronal resonance response in visual areas to 17 Hz flicker stimulation (or to nearby flicker frequencies, see [19] for stimulation between 1–100 Hz), i.e. there is no evidence for 17 Hz entrainment in visual areas. Secondly, 17 Hz does not correspond to the frequency of a known (inhibitory) rhythm of the visual system, further arguing against an entrainment account. Thirdly, in keeping with an upper dynamic limit is the fact that with only 4 entrainers, 17 Hz-cueing only lasts 176 ms (from entrainer 1 to 4), leaving arguably not enough time for the attentional system to keep up with the speed of presentation. We therefore conclude that the present result of failed attentional cueing at 17 Hz is more likely due to the attentional benefit breaking off at an upper limit than any other mechanism. It does remain unclear whether this limit reflects a fundamental (absolute) upper limit of the attention system, or more likely, whether it depends on the number of entrainers and the particular paradigm, as attention allocation may become more accurate and effective when more than four entrainers are employed (see [44]).

In future work, it would be of interest to delineate in more detail the exact constraints of rhythmic attentional cueing benefits through more detailed parametric manipulations, involving also frequencies above 17 Hz.

### Limitations and caveats

The most likely collective account of our data set is that entrainment of brain rhythms in the alpha band contributed to our results, because it can explain both alpha-specificity and alpha-periodicity of behavioral effects (experiment 1 and 2) in light of the link to posterior alpha oscillations (experiment 2b). Yet, although less likely, alternative explanations should be considered.

Since the delays between cues within the trains as well as between the last entrainer and visual target were different across frequencies of cueing (by design), we need to consider whether the results of experiment 1 (i.e. alpha-specificity) could have resulted from differential forward masking effects across conditions, differential attentional blinks associated with the rapid serial visual presentation per frequency, or differential apparent motion effects. First, forward masking effects (due to the last entrainer masking the visual target) may differentially interfere with perception depending on presentation frequency, as the cue-target delay (mask-target SOA) does affect the strength of masking [45]. Yet, since in the motion entrainment condition visual targets were not in the same location as the visual entrainers (the potential masks) and since motion entrainment did not differ from flicker entrainment in terms of frequency effects (no interaction between Rhythmic Cueing Type and Frequency of cueing), forward masking seems unlikely to explain our results. Second, the attentional blink may differentially affect perception across presentation frequencies because it also depends on SOA [46]. Yet, such an explanation would be difficult to reconcile with the pattern of cueing effects we observe across frequencies; in particular with the absence of benefits at two (5.3 Hz and 10.6 Hz) frequencies, but the intermediate frequency (i.e. 7.1 Hz) being unaffected. Third and in analogy to the above, although apparent motion depends on stimulation frequency [47], our pattern of findings with breakdowns of cueing at 5.3 Hz and 10.6 Hz but not at 7.1 Hz seems difficult to explain in this context. We therefore conclude that our findings are best interpreted in terms of entrained alpha-oscillations.

A limitation of our experiments is that we did not record MEG simultaneously with task performance. This means that we cannot directly relate the fluctuations in behavioural performance to brain oscillations, although we would expect perception and underlying alpha-oscillations to fluctuate in counter-phase at the time when the corresponding visual input signal reached the visual areas, because perception should peak at troughs of the underlying alpha oscillation [39]. In addition, this means that the postulated mechanism of alpha phase alignment explaining our results, although parsimonious and in line with previous work (see above and Introduction), is supported by our data only indirectly. A recent study by Mathewson et al. [21] however provides empirical support for this postulation. These authors measured electroencephalography (EEG) while rhythmic trains of visual stimuli preceded a visual target. Behaviorally, targets in phase with rhythmic trains were detected more often than targets out of phase. The EEG showed phase-alignment of alpha oscillations to the rhythmic train, and this increase in phase-locking predicted the increase in target discrimination when targets were in phase. These results demonstrate that rhythmic stimulation at alpha frequency could indeed align alpha oscillations which might be functionally relevant for visual performance [10], [14], [21], [37], supporting the interpretation of our findings here. In contrast to our findings, individual peak alpha frequencies (in resting state EEG) did not correlate to any of the behavioral measures in Mathewson et al. [21]. Also, the experimental design and behavioral analysis did not allow a direct evaluation of whether behavior oscillated at alpha frequency, and whether entrainment effects are frequency- and location-specific. Our results and those described in Mathewson et al. [21] are therefore highly complementary. Taken together, they strongly support the hypothesis that rhythmic visual stimulation phase-aligns intrinsic alpha oscillations that are directly relevant for successful visual perception.

### Alpha phase alignment by non-retinotopic mechanisms of entrainment?

One last aspect of our results that affords further consideration involves the spatial configuration of our stimuli in the flicker versus motion entrainment condition. The entrainers of the flicker condition are all positioned in the same visual field location. This is not the case for the motion condition, yet the results in these two conditions are not different (experiment 1). Despite the entrainers of the motion condition moving across visual field positions, the cueing benefit breaks down at 10.6 Hz independently of cueing type (flicker versus motion). This may indicate that entrainment of alpha-oscillations extends to non-retinotopic mechanisms. Indeed, attention research showed that alpha oscillations are not only up- or down-regulated by spatial attention in a retinotopically specific manner (as shown in [24], [28]) but also in areas of the ventral versus dorsal stream as a function of attention to ventral-stream versus dorsal-stream visual features (colour versus motion) [48]. This indicates that perceptually relevant alpha-oscillations are not restricted to retinotopic early visual cortex, but extend to higher-order visual areas. Accordingly, it is conceivable that these oscillations may also be entrained by appropriate frequency tagging of the relevant visual feature (i.e. presentation of apparent motion stimuli at alpha frequency for entrainment of alpha-oscillations in motion areas).

Finally, our result of cyclic patterns in perception that are locked to visual cues (experiment 2) is reminiscent of a recent finding by Landau and Fries [6]. Landau and Fries [6] showed that one single visual event in one hemifield can lead to phase-locked oscillations in visual performance in that hemifield, with visual performance in the opposite hemifield cycling in anti-phase. These results were interpreted to reflect rhythms of covert exploration (attentional scanning) after phase-locking of attention to the time point and visual position of the visual event. Our results are difficult to interpret in this context because they were obtained in a different frequency band, using a different paradigm and a lower temporal sampling rate of visual task performance. The phase-locking in our experiment may therefore not reflect correlates of attentional scanning (covert exploration behavior), but rather be best explained by phase-reset of oscillations generated in visual areas. This interpretation is supported by our finding that the cycles in visual task performance related to the individual alpha frequencies obtained from MEG.

### Conclusions

Our results demonstrate alpha-specificity and alpha-periodicity of behavioural effects to rhythmic cueing paradigms, correlating with brain oscillations as measured by MEG. They reveal the functional relevance of intrinsic alpha-oscillations in successful visual perception, and demonstrate that these oscillations can be controlled (and thus studied) by rhythmic visual stimulation at intrinsic frequencies.
